# Phylogenetic Analysis of an HIV Outbreak in a Dialysis Unit at a Tertiary Care Hospital in Multan, Pakistan

**DOI:** 10.3390/v18030318

**Published:** 2026-03-04

**Authors:** Syed Faisal Mahmood, Hasnain Javed, Ayesha Shahbaz, Nida Farooqui, Amna Rafique, Zainab Umar, Syed Hani Abidi

**Affiliations:** 1 Department of Medicine, Aga Khan University, Karachi 74800, Pakistan; faisal.mahmood@aku.edu (S.F.M.); ayeshashahbaz.k@gmail.com (A.S.); zainab.umar24@alumni.aku.edu (Z.U.); 2 Punjab AIDS Control Program, Lahore 54000, Pakistan; hasnain_javed@hotmail.com (H.J.); dramnarafiq44@gmail.com (A.R.); 3 Department of Biomedical Sciences, Nazarbayev University School of Medicine, Astana 010000, Kazakhstan; nidafarooqui95@gmail.com

**Keywords:** HIV-1, outbreak, point-source, phylogenetics

## Abstract

Background: In Pakistan, the number of Human immunodeficiency virus (HIV) cases is increasing significantly, attributed to risk factors such as injection drug use, sexual transmission, etc. However, transmission through hemodialysis units is not well documented. In 2024, an outbreak of HIV cases in Multan, Pakistan, drew alarm from local health authorities due to reports linking it to a large public hospital in South Punjab. Here, we report the molecular epidemiological investigation of the outbreak. Methods: Twenty-five hemodialysis patients identified during the outbreak were enrolled. Blood samples were subjected to DNA extraction and polymerase chain reaction (PCR) amplification. Phylogenetic analysis was conducted using the maximum-likelihood approach in IQ-TREE. For dating phylogenetics, a maximum clade credibility tree (MCC) was constructed using the BEAST tool. The MCC tree was constructed using the Bayesian Skyline model with an uncorrelated lognormal relaxed clock. The VESPA program was used to identify amino acid signatures unique to outbreak sequences compared with Pakistani reference sequences. Results: A total of 25 patients (identified as part of the HIV outbreak) were enrolled. 96% (24 out of 25) also tested positive for Hepatitis C, while none tested positive for Hepatitis B. The age range of patients in the study was 23 to 72 years (median age: 44.88 years). In terms of gender distribution, 13 out of 25 were male. All the sequences were identified as HIV subtype CRF02_AG. Phylogenetic analysis revealed that Multan sequences formed a well-supported monophyletic cluster, indicating shared recent origin. Signature pattern analysis identified a unique molecular fingerprint at 26 nucleotide positions, whereas molecular dating placed the emergence of the cluster between 2023 and 2024, consistent with the outbreak timing. Conclusions: Findings provide biologically plausible evidence of a point-source HIV outbreak linked to lapses in infection prevention and control practices at the hemodialysis unit.

## 1. Introduction

Chronic hemodialysis (HD) remains a mainstay of life-saving treatment for patients with end-stage renal disease (ESRD). However, the layout of most hemodialysis units predisposes to opportunities for transmission of nosocomial infections [1]. In particular, the transmission of blood-borne pathogens is attributed to the percutaneous exposure to blood from infected patients, which can occur during hemodialysis [2].

The nosocomial spread of Hepatitis B virus (HBV) and Hepatitis C virus (HCV) in hemodialysis units has been well documented [3]. However, the risk of nosocomial spread of Human Immunodeficiency Virus (HIV), which shares similar routes of transmission to these bloodborne pathogens, is only 0.3% [3]. Recent studies have challenged the idea that hemodialysis remains a low-risk setting for HIV transmission, as findings from a retrospective case–control study conducted by the Centers for Disease Control and Prevention (CDC) suggest that percutaneous injuries involving large volumes of blood or higher HIV titers surpass documented risk estimates [2]. Studies conducted in hemodialysis units in Colombia [4] and Saudi Arabia [5] mirrored these findings.

The HIV epidemic in Pakistan is complex and understudied. Since the first report of HIV-1 in 1987 [6], the number of HIV cases had proportionally increased to about 210,000, including people of all ages, in 2021 [7]. HIV is highly prevalent in three high-risk groups, namely, people who inject drugs (PWID), transgender sex workers (also known as Hijra sex workers (HSW)), and men who have sex with men (MSM) [7,8,9,10], where prevalence is around 38–40% [11,12], 11% and 7.5%, respectively [11]. Awareness regarding HIV status is extremely low, where only 36,000 of an estimated 0.16 million People Living with HIV (PLWH) know that they are HIV positive [13]. Furthermore, treatment coverage in Pakistan is also very low, and only 58,622 individuals are receiving HIV treatment [13].

In Pakistan, there have been eight documented HIV outbreaks in the past two decades, including the 2019 outbreak in Sindh province, where more than 900 individuals, mostly children, have been identified as HIV positive, making this outbreak the largest HIV outbreak in children in the world. However, transmission through hemodialysis units is not well documented.

In 2024, an outbreak of HIV cases in Multan, Pakistan, drew alarm from local health authorities, due to reports linking it to a large public hospital in South Punjab [14]. A committee subsequently created to investigate this alarming development confirmed an outbreak of HIV in the hospital’s nephrology unit. To assess whether this was a point source outbreak or an overall increase in HIV incidence in the population, a collaborative molecular investigation was launched to perform phylogenetic analysis on the HIV sequences obtained from the patients involved in the outbreak.

This study presents the findings of the molecular analysis conducted on the HIV outbreak in Multan.

## 2. Materialand Methods

### 2.1. Study Design and Samples

This was a cross-sectional study. The outbreak occurred between October and December 2024 at a dialysis unit in a tertiary care hospital in Multan, Pakistan. This hospital serves a large population across South Punjab. Twenty-five hemodialysis patients were identified in an HIV outbreak. After obtaining written informed consent, 5 mL of blood was collected from each patient for genotyping. Simultaneously, a questionnaire was administered to collect patient data. Patient data were entered into REDCap version 14.0, exported to Microsoft Excel, and analyzed using descriptive statistics. This study was approved by the Aga Khan University Ethical Review Committee (2024-7573-29880).

### 2.2. DNA Extraction, PCR, and Sequencing

Samples were transferred to Aga Khan University, Karachi, at 4 °C, where they were used for DNA extraction with the Qiagen DNA extraction kit (Qiagen, Hilden, Germany), following the manufacturer’s protocol. The DNA was purified and eluted into a final volume of 100 µL for further analysis. DNA samples were subjected to polymerase chain reaction (PCR) amplification using the following primers for the first round of PCR: upstream primers F1a: 5’-TGAARGAITGYACTGARAGRCAGGCTAAT-3’ and F1b: 5’-ACTGARAGRCAGGCTAATTTTTTAG-3’; downstream primer RT-R1: 5’-ATCCCTGCATAAATCTGACTTGC-3’. The primers used for the second round of PCR were: upstream primer PRT-F2: 5’-CTTTARCTTCCCTCARATCACTCT-3’ and downstream primer RT-R2: 5’-CTTCTGTATGTCATTGACAGTCC-3’. The total amplicon size was approximately 1197 bp, targeting the HIV *protease* (PR) and *reverse transcriptase* (RT) gene [15]. A positive control was included in each PCR run to ensure the validity of the results. The reaction conditions for the PCR were: 94 °C for 5 min; 35 cycles of 94 °C for 15 s, 52 °C (first round) and 54 °C (second round) for 20 s, and 72 °C for 2 min; 72 °C for 10 min; and holding temperature of 4 °C using a Taq PCR master mix (Applied Biological Materials Inc, Richmond, BC, Canada, Cat. No. G888). Finally, the amplified PCR products were sequenced by Eurofins Scientific (Luxembourg City, Luxembourg), and the sequences were deposited in NCBI GenBank and assigned accession numbers PX057081–PX057098.

### 2.3. HIV Subtyping, Sequence Alignment, Phylogenetic, and Drug-Resistance Mutation Analysis

HIV subtyping was performed using the REGA HIV-1 subtyping tool version 3.0 (http://dbpartners.stanford.edu:8080/RegaSubtyping/stanford-hiv/typingtool/; accessed on 8 February 2026), which uses phylogenetic analysis and a bootscanning-based method to determine subtypes from genomic sequences [16,17]. All the sequences were identified as subtype CRF02_AG.

The nucleotide sequences were used to generate the reference dataset, comprising CRF02_AG sequences closely matching the outbreak sequences, using a Basic Local Alignment Search Tool (BLAST+ 2.17.0) approach described previously [18,19]. The Multan sequences (*n* = 18) were aligned with previously amplified Pakistani CRF02_AG sequences (*n* = 27) and global HIV CRF02_AG sequences (*n* = 16) using MEGA 7.0 software, and the alignment was then used to construct a maximum-likelihood (ML) phylogenetic tree in IQTREE version 3.0.1 (https://iqtree.github.io/; accessed on 28 February 2025) with the following parameters: nucleotide model: GTR+I; node support: Shimodaira-Hasegawa approximate likelihood ratio test (SH-aLRT) statistics, where nodes with SH-aLRT values of 90 or higher were considered significant [20].

For dating phylogenetics, an MCC tree was constructed using BEAST. The MCC tree was constructed using the Bayesian Skyline model with an uncorrelated lognormal relaxed clock and under the GTR+Γ4+I substitution model [21]. BEAST runs of 100 million generations, sampling every 10,000th iteration, and discarding the first 10% of samples as burn-in, were performed. The run gave an effective sample size (ESS) ≥ 200, which was analyzed using Tracer version 1.7.0 software [22].

Drug resistance mutation (DRM) analysis was performed using the DRM analysis tool available on the Stanford Drug Resistance Database [23].

### 2.4. Viral Signature Pattern Analysis

The VESPA (http://www.hiv.lanl.gov/content/sequence/VESPA/vespa.html; accessed on 8 February 2026) programme was used to identify amino acid signatures unique to outbreak sequences, compared withPakistani reference sequences [24,25].

### 2.5. Statistical Analysis

Statistical significance was assessed using Fisher’s exact test in SPSS version 12.0. Statistical significance was defined as *p* < 0.05.

## 3. Results

### 3.1. Study Participants

The profile of study participants is described in Table 1. A total of 25 patients were enrolled as part of the HIV-1 outbreak. 96% (24 out of 25) also tested positive for Hepatitis C, while none tested positive for Hepatitis B. Out of twenty-five samples, 18 samples were successfully amplified. The age range of patients in the study was 23 to 72 years (median age: 44.88 years). In terms of gender distribution, 13 out of 25 (52%) were male. Seventeen of twenty-five patients were married. None of the patients reported multiple partners. The average duration of dialysis was 4.2 years, with three individuals on dialysis for more than 10 years. Hemodialysis frequency was reported as 2–3 times per week for all patients. Furthermore, 92% of infected patients (22 of 24) had an arteriovenous fistula (AVF), the most common type of vascular access. No patients reported previous vascular access infections or intravenous (IV) drug use. Partner gender (male, female, or transgender) and occupational risk factors were not determined.

### 3.2. Phylogenetic and Drug Resistance Mutation Analysis

**Figure 1:**
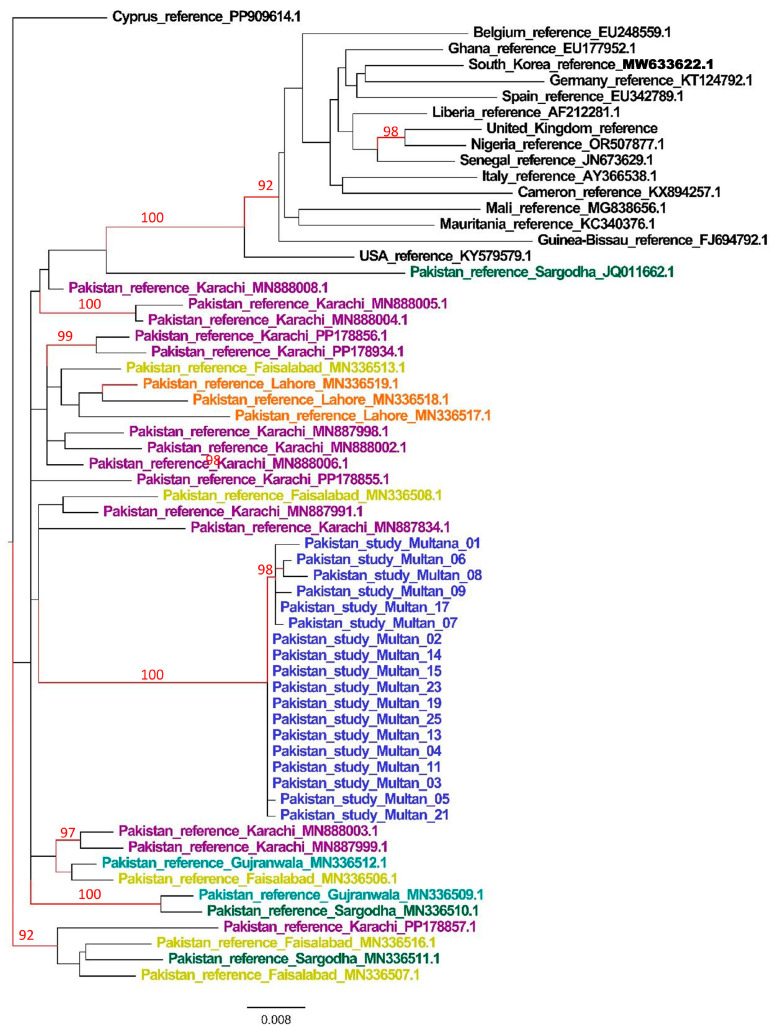
ML tree of Multan HIV outbreak sequences. The study sequences are shown in blue, while the reference sequences are shown in black. Sequences uencesin purple, orange, gteal, blue, and yellow represent Pakistani reference sequences from Karachi, Lahore, Sargodha, Gujranwala, and Faisalabad, respectively. tree bar indicates the number of substitutions per siten site. Nodes with significant SH-aLRT (≥90) values are shown in red.

For phylogenetic/MCC analysis, outbreak sequences (subtype CRF02_AG) along with previously amplified Pakistani and global CRF02_AG sequences (matching the outbreak sequences in BLAST search) were used. The phylogenetic analysis revealed that the Multan sequences formed a well-supported monophyletic cluster, with an SH-aLRT node support > 90%, suggesting a common origin (Figure 1). This cluster was embedded within the Pakistani cluster, comprising sequences from Karachi and Faisalabad, indicating that the sequences originated within Pakistan rather than outside the country.

The MCC tree (constructed using outbreak and reference CRF02_AG sequences) further confirmed the clustering pattern for Multan sequences, with a node age of 2024 (95%HPD upper-lower: 2023–2024; Figure 2). This is consistent with an outbreak or a rapid spread from a single source.

DRM analysis identified major DRMs in one sample: K70R, associated with resistance to zidovudine (AZT), and V108I, associated with resistance to doravirine (DOR) and efavirenz (EFV).

### 3.3. Signature Mutations in Outbreak and Local Sequences

Signature mutation analysis identified 26 positions as unique (*p* < 0.001) in outbreak sequences compared with Pakistani reference sequences. Out of 26, 14 positions contained non-synonymous nucleotide changes, while 12 positions contained synonymous changes (Table 2).

## 4. Discussion

This study presents the findings of the molecular analysis conducted on the HIV outbreak in Multan.

Phylogenetic analysis of HIV sequences was used to provide biologically plausible evidence of a point-source HIV outbreak originating at a hemodialysis centre. The use of phylogenetic tools to provide robust evidence for point-source outbreaks is well documented. During the 2014 West Africa Ebola virus outbreak, Gire et al. used whole-genome phylogenetic analysis and molecular clock dating to show that the epidemic arose from a single zoonotic spillover into humans, followed by sustained human-to-human transmission [26]. Similarly, the 2003 outbreak of Legionnaires' disease in Hereford, UK, was investigated by Reuter et al., who found that outbreak-related clinical isolates were nearly identical to cooling-tower isolates and clearly distinct from unrelated strains, confirming a single environmental point source [27]. In our study, phylogenetic analysis of HIV sequences demonstrated that the Multan sequences formed a well-supported monophyletic cluster, indicating a shared recent origin [28]. Molecular dating placed the emergence of this cluster between 2023 and 2024, which is consistent with the timing of the outbreak.

Lapses in infection prevention and control (IPC) are hypothesized to play a pivotal role in the Multan outbreak. IPC breaches, such as the reuse of contaminated equipment and inadequate surface decontamination, have been linked to nosocomial outbreaks of blood-borne diseases worldwide. A systematic review by Sacuk et al. found that most blood-borne infection outbreaks in hemodialysis units were caused by inadequate surface disinfection, followed by hand-hygiene violations [29]. Velandia et al. investigated the 1993 HIV outbreak in Colombia, concluding that suboptimal infection control practices, such as cross-contamination of access needles in a shared, low-level disinfectant, constituted the mode of patient-to-patient HIV transmission [4]. Similarly, HIV transmission in hemodialysis units in Saudi Arabia has been linked to IPC lapses, including single-use medication on multiple patients, contaminated gloves, and inadequately disinfected equipment [5]. After investigating the public hospital and hemodialysis centre where the Multan outbreak occurred, a probe body found evidence of violation of standard operating procedures (SOPs) and negligence by hospital staff.

Pakistan has seen multiple HIV outbreaks since its first documented case in 1987. The earliest large-scale outbreak was reported in Larkana in the early 2000s among people who inject drugs (PWIDs), followed by several community and healthcare-related outbreaks across Punjab and Sindh. Notably, pediatric outbreaks in Ratodero (2019) and Kot Imrana (2018–2019) were linked to unsafe medical practices, including the reuse of contaminated injection equipment [30]. In October 2016, 50 HIV cases were reported at a dialysis unit in Larkana, Sindh [31]. An investigation carried out by the National AIDS Control Programme (NACP) found evidence of poor IPC practices, a lack of dedicated dialysis equipment for infected patients, and patients purchasing blood products from unregulated blood banks and laboratories. The Multan outbreak aligns with this broader national pattern, reinforcing concerns regarding IPC practices in high-risk healthcare settings such as hemodialysis units.

A key finding among the Multan outbreak cohort was the high rate of blood transfusions, with 88% reporting a prior transfusion history and 30% receiving two or more transfusions per year [32]. Sacuk et al. [29] report that transfusion-associated transmission is almost exclusively seen in low-income settings. This can be explained by WHO data showing that blood donations in high-income countries are almost universally screened. In contrast, in middle- and low-income countries, screening is only 83% and 76%, respectively. Lack of robust hemovigilance systems, suboptimal blood transfusion practices, and population unawareness contribute to these challenges. This is in line with documented reports of unsafe blood transfusion practices in Pakistan, which have been linked to high rates of transfusion-transmitted infections (TTIs) [33]. Thus, suboptimal blood transfusion practices may also be a potential route of HIV transmission amongst our cohort.

Preventing HIV transmission in haemodialysis units requires the rigorous application of standard precautions. Hand hygiene, appropriate use of personal protective equipment, and strict sharps safety protocols form the foundation of these measures [34]. Notably, dedicated dialysis machines are not required for HIV-positive patients, provided disinfection protocols are strictly observed between patient shifts [35]. Sacuk et al. [29] found that most blood-borne infection outbreaks in haemodialysis units were caused by inadequate surface disinfection, followed by hand-hygiene violations, reinforcing the importance of pre- and post-session decontamination of dialysis station surfaces. Medication vials should be discarded after single use, with medications prepared centrally away from the patient treatment area. Regarding surveillance, all patients initiating haemodialysis should undergo HIV screening, with enhanced monitoring reserved for those with identifiable risk factors. Any new HIV case identified within a unit should prompt an immediate outbreak response, incorporating expert review, testing of all potentially exposed patients, and a thorough assessment of infection control practices. Finally, all blood products administered should be screened for HIV, ideally using nucleic acid amplification tests.

We found DRMs K70R, associated with resistance to zidovudine (AZT), and V108I, associated with resistance to doravirine (DOR) and efavirenz (EFV), in one sample.

The application of signature pattern analysis is also instrumental in tracing transmission and genetic variation in HIV [36]. Our mutation analysis identified 26 unique nucleotide positions in the 2025 Multan HIV cluster, including 14 non-synonymous and 12 synonymous amino acid changes. The non-synonymous changes may lead to functional alterations, while the synonymous changes indicate a silent marker for a possible evolutionary link in Pakistan [37]. Identifying such unique patterns remains a critical pillar of HIV surveillance, underscoring the need for this approach in both outbreak investigation and clinical treatment strategies.

Our study had certain limitations. A smaller sample size limits the generalizability of our findings to other dialysis populations within Pakistan. Additionally, due to the retrospective nature of data collection, information such as exact transfusion dates and other relevant patient history points could not be extricated. Key information on the hospital’s IPC practices was also exempt, limiting evidence-based critique of IPC gaps. Finally, while the phylogenetic analysis strongly suggests a recent common source of infection, the precise point of origin or mechanism of transmission cannot be definitively established without further epidemiological tracing.

In conclusion, although phylogenetic analysis indicates a recent common source of infection, the exact origin or transmission pathway remains unclear without further epidemiological investigation. The findings emphasize the role of hemodialysis units in HIV transmission and call for strategies to enhance patient safety and prevent future outbreaks.

## Figures and Tables

**Figure 2:**
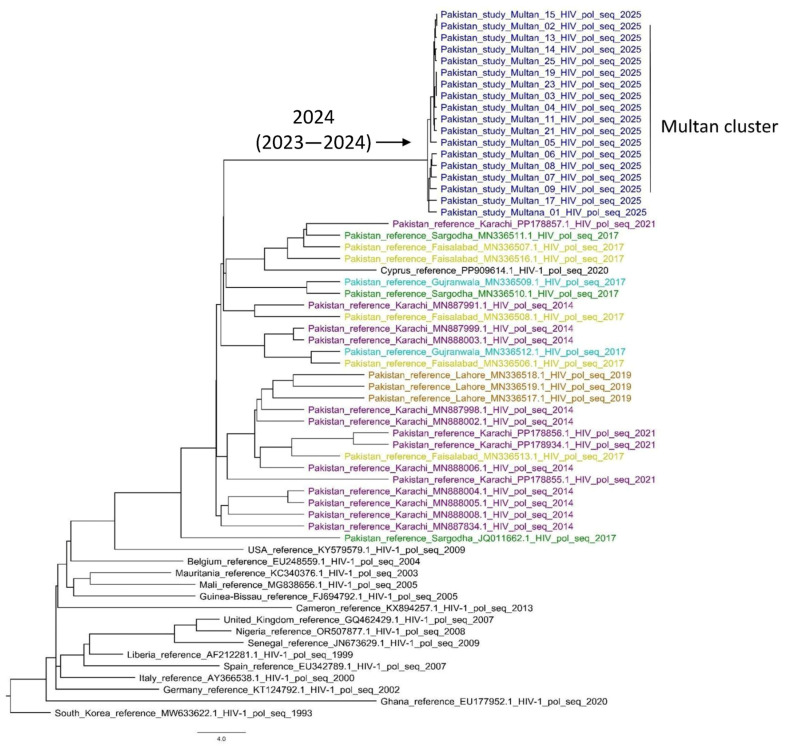
MCC tree of Multan HIV outbreak sequences. The outbreak sequences are shown in blue, while the reference sequences are shown in black. Sequences in purple, orange, green, teal, and yellow represent Pakistani reference sequences from Karachi, Lahore, Sargodha, Gujranwala, and Faisalabad, respectively.

**Table 1:** Baseline characteristics and risk exposures of participants included in the Multan HD outbreak investigation.

Characteristic	n (%) or Median (IQR)
Age, years	44 (33–55)
Sex	
Male	13 (52.0)
Female	12 (48.0)
Marital status	
Married	17 (68.0)
Single	4 (16.0)
Widowed/Widower	1 (4.0)
Unknown	3 (12.0)
Hepatitis B status (HBsAg)	
Negative	25 (100.0)
Hepatitis C status	
Positive	24 (96.0)
Negative	1 (4.0)
Risk exposures	
History of intravenous drug use	0 (0.0)
History of blood transfusion	22 (88.0)
History of IV/IM medication injections	5 (20.0)

**Table 2:** Signature mutations in outbreak and local sequences. The table shows nucleotide variants in the outbreak sequences relative to the Pakistani reference sequences. For each position, the study nucleotide with its frequency (1.0 = fixed) and the reference nucleotide are shown. Column 2 indicates the changes in nucleotide as nonsynonymous (NS) or synonymous (S). The last column shows the corresponding amino acid changes in the outbreak sequences compared to the Pakistan reference sequences.

Position	Type	Nucleotide (Amino Acid) in Outbreak Sequences	Nucleotide (Amino Acid) in Reference Sequences	Frequency (Outbreak Sequences)	Frequency (Reference Sequences)
5	NS	A (Ala)	G (Gly)	1	0.163
87	NS	G (Gly)	A (Ala)	1	0.047
145	NS	G (Gly)	C (Cys)	1	0.023
195	NS	C (Cys)	T (Phe)	1	0.023
225	NS	A (Ala)	G (Gly)	1	0.14
252	NS	C (Cys)	T (Phe)	1	0
264	NS	C (Cys)	T (Phe)	1	0
309	NS	A (Ala)	C (Cys)	1	0.023
366	S	T (Phe)	T (Phe)	1	0.326
370	NS	G (Gly)	A (Ala)	1	0.186
408	S	G (Gly)	G (Gly)	1	0
453	NS	G (Gly)	A (Ala)	1	0.07
549	NS	A (Ala)	G (Gly)	1	0.233
579	S	C (Cys)	C (Cys)	1	0
609	S	T (Phe)	T (Phe)	1	0.07
702	S	T (Phe)	T (Phe)	1	0.023
744	S	C (Cys)	C (Cys)	1	0.14
789	NS	G (Gly)	A (Ala)	1	0.419
852	NS	G (Gly)	T (Phe)	1	0.372
867	NS	A (Ala)	G (Gly)	1	0.349
883	S	C (Cys)	C (Cys)	1	0.163
894	S	A (Ala)	A (Ala)	1	0.023
909	S	T (Phe)	T (Phe)	1	0.093
912	S	A (Ala)	A (Ala)	1	0.093
918	S	C (Cys)	C (Cys)	1	0.023
948	S	G (Gly)	G (Gly)	1	0.326

## Data Availability

All data are available in the manuscript. The sequences generated for this study were deposited in NCBI GenBank and assigned accession numbers PX057081–PX057098.
